# The Effects of the Digital Platform Support Monitoring and Reminder Technology for Mild Dementia (SMART4MD) for People With Mild Cognitive Impairment and Their Informal Carers: Protocol for a Pilot Randomized Controlled Trial

**DOI:** 10.2196/13711

**Published:** 2019-06-21

**Authors:** Peter Anderberg, Pilar Barnestein-Fonseca, Jose Guzman-Parra, Maite Garolera, María Quintana, Fermin Mayoral-Cleries, Evi Lemmens, Johan Sanmartin Berglund

**Affiliations:** 1 Department of Health Blekinge Institute of Technology Karlskrona Sweden; 2 Research Unit, La Unidad de Gestión Clínica de Mental Health Instituto de Investigación Biomédica de Málaga Hospital Regional Universitario Málaga Malaga Spain; 3 Brain, Cognition and Behavior - Clinical Research Consorci Sanitari de Terrassa Barcelona Spain; 4 University Colleges Leuven-Limburg Genk Belgium

**Keywords:** dementia, mild cognitive impairment, tablet, app, carer, eHealth, mHealth

## Abstract

**Background:**

Many countries are witnessing a trend of growth in the number and proportion of older adults within the total population. In Europe, population aging has had and will continue to have major social and economic consequences. This is a fundamentally positive development where the added life span is of great benefit for both the individual and the society. Yet, the risk for the individual to contract noncommunicable diseases and disability increases with age. This may adversely affect the individual’s ability to live his or her life in the way that is desired. Cognitive conditions constitute a group of chronic diseases that predominantly affects older people. Recent technology advancements can help support the day-to-day living activities at home for people with cognitive impairments.

**Objective:**

A digital platform (Support Monitoring and Reminder for Mild Dementia; SMART4MD) is created to improve or maintain the quality of life for people with mild cognitive impairment (PwMCI) and their carers. The platform will provide reminders, information, and memory support in everyday life, with the purpose of giving structure and lowering stress. In the trial, we will include participants with a diagnosed neurocognitive disorder as well as persons with an undiagnosed subjective memory problem and cognitive impairment, that is, 20 to 28 points on the Mini-Mental State Examination.

**Methods:**

A pragmatic, multicenter RCT is being conducted in Spain, Sweden, and Belgium. The targets for recruitment are 1200 dyads—split into an intervention group and a control group that are in usual care. Intervention group participants will be provided with a data-enabled computer tablet with the SMART4MD app. Its core functionalities, intended to be used daily at home, are based on reminders, cognitive supporting activities, and sharing health information.

**Results:**

Inclusion of participants started in December 2017, and recruitment is expected to end in February 2019. Furthermore, there will be 3 follow-up visits at 6, 12, and 18 months after the baseline visit.

**Conclusions:**

This RCT is expected to offer benefits at several levels including in-depth knowledge of the possibilities of introducing a holistic multilayered information and communication technology solution for this group. SMART4MD has been developed in a process involving the structured participation of PwMCI, their informal carers, and clinicians. The adoption of SMART4MD faces the challenge of this age group’s relative unfamiliarity with digital devices and services. However, this challenge can also be an opportunity for developing a digital device tailored to a group at risk of digital exclusion. This research responds to the wider call for the development of digital devices which are accessible and affordable to older people and this full scale RCT can hopefully serve as a model for further studies in this field.

**Trial Registration:**

ClinicalTrials.gov NCT03325699; https://clinicaltrials.gov/ct2/show/NCT03325699

**International Registered Report Identifier (IRRID):**

DERR1-10.2196/13711

## Introduction

### Background

The number of older adults in the population as well as their proportion within the total population are increasing in many parts of the world. In Europe, population aging has had and will continue to have major social and economic consequences. This is a fundamentally positive development where the added life span is of great benefit for both the individual and the society. Many older people are able to support themselves and continue to make important contributions to society. Yet, the risk for the individual to contract noncommunicable diseases and disability increases with age. This may impair the ability of the individual to live his/her life in the way that is desired. It also exerts pressure on health services and support systems for older people.

Cognitive conditions such as mild cognitive impairment (MCI) and dementia constitute a group of chronic diseases that predominantly affects older people [1]. The general increase in life span causes the number of people with MCI [2] to increase; people with dementia in Europe alone is estimated to reach 13.4 million by 2030. The challenge of ensuring a good life for all is likely to remain formidable [3]. Health care policies are focused on extending the ability of older people to continue to live independently as one way of meeting these challenges. As medical treatment options currently remain centered on symptomatic treatment, the use of technology [4] and specifically information and communication technologies (ICTs) is suggested as a way to support function and maintain a good life [5,6]. For people with neurocognitive disorders, a good quality of life (QoL) means that they experience a certain level of independence and self-management of daily-life activities, which may be promoted by using technology.

It is important to be aware of the heterogeneity in technology use abilities [7-9]. Involving end users in design and development of technology is especially important for the development of usable technology [10-12].

Cognitive problems affect both the individual as well as their surroundings [13,14]. Living together with and/or caring for a PwMCI or dementia can compromise the informal carer’s own well-being and health because of a feeling of being overloaded in the caregiving role [15-17].

The central role the informal carers play also accentuates the importance of the well-being of the informal carers themselves, and provision of support to the informal carers is therefore essential [18]. This is also an important target for the development of new supportive technologies. For example, people with dementia often experience disorientation and thus tend to get lost while they are outside on their own, causing stress for the caregiver. According to a pilot study by Pot et al [19], global positioning system tracking reduces feelings of worry when being alone outside, both in people with dementia as well as their caregivers. The Rosetta system integrated 3 ICT systems to support daily functioning (eg, social contact, memory, and activities) and to detect changes in daily behavior and identify emergency situations. Using this system, feelings of safety and security improved QoL in people with dementia and their carers [12].

Many devices and digital apps to support the day-to-day living activities at home for people with neurocognitive disorders have been developed during the recent years. However, most solutions focused on cognitive impairment training or support and safety for people living alone. Most of them are single apps or devices, but few were integrated into a single solution or product [20,21].

A recent study by Yousaf et al [22] categorized mobile health apps for dementia into apps for cognitive training and daily living (eg, word games, videos, and music), screening (eg, clock drawing), health and safety monitoring (eg, fall detection using installed cameras, alarms, and emergency help), leisure and socialization (eg, media for reminiscence therapy), and navigation (eg, tracking). Similarly, Meiland et al [11] looked at 3 fields of technologies for this group: (1) support with managing everyday life, (2) support with participating in pleasurable and meaningful activities, and (3) support with dementia health and social care provision. The study highlighted the importance of both inclusion of the group in the development of technology as well as the need for more comprehensive studies and higher quality evaluation of technology usability. Furthermore, the early introduction of technology in a degenerative cognitive process, or preferably before, may be an important factor [23,24].

The importance of including QoL as an outcome measure in dementia trials has been identified by the World Federation of Biological Psychiatry’s Old Age Taskforce [25] as well as others in the field. QoL is multifactorial; therefore, besides “the individual’s perception of their position in life in the context of culture and value systems in which they live, and in relation to their goals, standards, and concerns” [26,27], QoL also encompasses physical and mental health, social relationships, and participating in activities. In this trial, we therefore aim to analyze QoL in a broad sense.

Few systematic reviews are available that investigate the effectiveness of interventions on the QoL of people with dementia [11]. Among those available, 1 systematic review found preliminary evidence that nonpharmacological interventions targeting both patients and their carers improved the QoL of persons with dementia living at home [28]. In contrast, a systematic review of pharmacological interventions to improve QoL and well-being in persons with dementia found that these interventions failed to show positive results in these domains [25]. Other relevant systematic reviews focused on the use of assistive technologies to improve QoL in older people in general [29,30]. These covered technologies, including the computer and the internet (ie, general ICT), robotics, sensors, telemedicine, medication management apps, mobile tracking devices, and video games. Regarding QoL in older people with dementia specifically, the review [29] identified 3 studies investigating general ICT and sensors. These studies suggested that the investigated technologies have the capacity to enhance QoL not only in people with dementia but also in those caring for them. It is important to note that most studies included in these reviews were small, not randomized, did not include or report QoL as a primary outcome measure, or did not include a valid QoL measure [29]. Altogether, they state a current lack of high-quality evidence that these interventions improve the QoL of persons with neurocognitive disorders and underline the need of large randomized controlled trials (RCTs).

Results from a randomized controlled pilot study were published using a computerized platform entitled “A technology platform for the Assisted living of Dementia elderly Individuals and their informal carers” (ALADDIN) [31]. The authors demonstrated that the use of ALADDIN enhanced the carers’ ability to care for a person with dementia by improving the QoL and reducing distress and carer burden.

MCI and dementia are used mostly as terms in this text. Today, these conditions are more correctly referred to as neurocognitive disorders, classified according to diagnostic criteria in the fifth edition of the Diagnostic and Statistical Manual of Mental Disorders. However, as the earlier concepts, to a large extent, are still used in the literature, we have chosen not to continually change terms at all instances in this paper.

### Aim

In this trial, we aim to create a digital platform (Support Monitoring and Reminder for Mild Dementia, SMART4MD) to improve or maintain the QoL for people with mild neurocognitive disorders and their carers. The platform will provide the individual with reminders, information, and memory support in everyday life with the purpose of giving structure and lowering stress. In the trial, we will include participants with a diagnosed neurocognitive disorder as well as persons with an undiagnosed subjective memory problem and cognitive impairment, that is, 20 to 28 points on the Mini-Mental State Examination (MMSE). We will refer to this group as persons with mild cognitive impairment (PwMCI) in the following text.

### Primary Objective

The research hypothesis is that use of the SMART4MD app over a period of 18 months can result in an improvement in the QoL of PwMCI.

The statistical null hypothesis is that the use of the SMART4MD platform produces no change in the mean total Health-related quality of life Alzheimer's disease QoL-AD score of PwMCI over 18 months.

The statistical alternative hypothesis is that the use of the SMART4MD platform produces a change in the mean total QoL-AD score of PwMCI over 18 months.

### Secondary Objectives

The secondary objectives also apply to the intervention group using the app over a period of 18 months. The objectives are as follows:

- To increase adherence to prescribed medication by 10% (all documented prescriptions)
- To reduce functional decline in PwMCI by 10% (as measured by Instrumental Activities of Daily Living)
- To monitor PwMCI’s attendance at health care appointments and admission to health care institutions
- To monitor the informal carers’ mental well-being as a strong determinant of caregiver burden and in turn of QoL of informal carers in the control and intervention group

## Methods

### Study Design

This is a pragmatic, multicenter RCT, and the Consolidated Standards of Reporting Trials (CONSORT) [32] guidelines will be followed. The ClinicalTrials.gov identifier is NCT03325699.

### Setting

This study will be performed in 3 European countries: Spain, Sweden, and Belgium and 4 participant centers, which are as follows: Consorci Sanitari de Terrassa (Catalonia, Spain), Servicio Andaluz de Salud (Andalusia, Spain), Blekinge Institute of Technology (Sweden), and University College Leuven-Limburg (Belgium).

### Participants

In total, 1200 participant dyads in 3 European countries (Spain, Sweden, and Belgium) will be included in the study. The participant dyads will comprise the PwMCI and his/her informal carer, with the PwMCI being the main participant. The participant will be selected by a nonprobabilistic consecutive sampling method. All participants will receive their treatment as usual (TAU) in their respective site, but the care of the intervention group will be improved with the support of the SMART4MD app.

### Sample Size

A total of 1200 dyads (PwMCI+carers) is the target for recruitment, divided over 2 groups: an intervention group, using the SMART4MD app, and a control group, not using the SMART4MD app. In this clinical trial, QoL is the primary outcome measure, measured by the QoL-AD total score [33]. This score is based on 13 four-point (1 to 4) items on a discrete visual analog scale. Recent papers [34] show the standard deviation (SD) of the total QoL-AD score approaches 7.

To detect a small effect size of 0.2 comparing 2 groups (intervention and control), using a 2-sided *t* test with a 5% statistical significance level, with a power of 80%, the minimum number of PwMCI required in each group is 394 (788 overall). If there would be a dropout rate of 20%, the number of PwMCI registered on the study would need to be 493 in each group (thus 986 overall).

On the other hand, one of our secondary objectives is to increase adherence to prescribed medication by 10% in the intervention group. If we consider that in people with dementia, the treatment adherence is between 44% and 99% [35,36], and assuming the less prevalence (44%), which we want to increase by 10%, with a power of 0.8 and a significance level of .05, we need 1174 subjects (20% dropouts included). Therefore, the sample size of this study is adequate to detect statistical and clinical differences between groups for the QoL-AD (primary outcome) and treatment adherence (a secondary outcome).

### Inclusion Criteria

A participant will be eligible for inclusion in this trial only if all of the following criteria are met:

- Participants score 20 to 28 points on the MMSE whether or not a diagnosed neurodegenerative disease is present;
- A professional assessment of the patient's own experience of memory problems over a substantial period of time (more than 6 months);
- Participants are older than 55 years;
- Participants are home care recipients;
- Participants have an informal carer;
- Participants take prescribed medication and are in charge of their own medication use;
- Participants have no specific conditions reducing their physical ability to use the app, for example, visual, hearing, or motor impairments.

### Exclusion Criteria (Persons With Mild Cognitive Impairment Only)

A participant will not be eligible for inclusion in this trial if any of the following criteria apply:

- Participants have a terminal illness with less than 3 years of expected survival;
- Participants score above 11 on the Geriatric Depression Scale (GDS-15) [37] or have another known significant cause of disease as an explanation for cognitive impairment such as abuse and other psychiatric diagnoses such as bipolar disorders, schizophrenia, and developmental disorders.

### Recruitment

Participants will be identified from a cohort of people with cognitive impairment that has been present for more than 6 months and who meet all the study eligibility criteria. Participants can be under primary care services as well as secondary care services, such as those who are being followed up in memory clinics, outpatient clinics, day hospitals or other components of specialist mental health care, geriatric medicine, and neurology services. Participants will also be identified from patient databases such as those integrated in the centers’ networks. The identification process will consist of screening using information gathered from medical notes, clinic records, and/or clinical consultations for initial eligibility based on inclusion criteria.

Each country will have a country specific recruitment plan that will be revised by the trial steering committee.

After a brief explanation of the study design and research goals, participants will be invited to participate in the study, and an appointment with the researchers will be made. Participants will be provided with all the information they need to make an informed decision via a participant information sheet. They will be given a cooling-off period of at least 24 hours between informally agreeing to participate in the study and being invited to formally consent in a meeting with the research team.

At the first visit, the researcher will explain the study in detail and answer any questions the patient or caregiver may have. The patient’s eligibility will be confirmed and their ability to consent will be assessed. Once consent is officially given by signing an informed consent form by all parties, the dyad will be randomized into either the intervention or the control group.

### Randomization

Enrolled dyads will undergo 1:1 block randomization by each study clinic performed by the Anglia Ruskin Clinical Trials Unit (ARCTU) to assign them to the intervention group or the control group. Randomization will be undertaken using an internet-based randomization system setup by ARCTU on the TENALEA system provided by FormsVision BV. The system stores the predetermined sequence of randomization; this list is not available to the investigators.

### Intervention: the Support Monitoring and Reminder Technology for Mild Dementia Platform

Our intervention strategy, which aims to improve the QoL of PwMCI, is based on the use of a single or multiple function that the SMART4MD app enables. The rationale behind and description of the functionalities of the SMART4MD app are presented in detail in a separate document (Multimedia Appendix 1). Briefly, participants in the intervention group will be provided with a data-enabled computer tablet preinstalled with the SMART4MD app. The tablet will be configured in such a way that it is not possible for participants to download any other app or software onto the tablet.

SMART4MD is a general electronic health app that has been adapted specifically for MCI through a structured process involving the participation of PwMCI and their informal carers and health care professionals. Focus groups were conducted with PwMCI and their informal carers, and interviews were conducted with health care professionals. The app can run both on tablet and mobile phone devices adopting the Android operating system but is specifically optimized for tablet devices as opposed to smaller screen mobile phone devices.

The core functionalities of the app are based on reminders (medication, appointments with health care providers, and meeting up with family and friends), cognitive supporting activities (clock, calendar, brain games, and photos) and optional status and health information sharing with family and informal carers (including mood and specific health problems such as headaches).

The app allows the participant to share health information to other people of their choice (relatives or research team) by email. The participant can select what kind of information he/she can share and to whom he/she wants to send this information.

An important feature of SMART4MD is its personalization facility: main users (PwMCI and informal carers) will be able to switch on/off or change various features and information-sharing possibilities.

The app is intended to be used daily at home, mainly by the PwMCI themselves, with the help of their informal carers when needed.

### Training and Support for App Users

The SMART4MD user training across the trial sites will be delivered in the most uniform way possible, minimizing bias. Support during the trial regarding the use of the app will be provided, and each center will nominate 1 individual to lead the support for participants recruited and assigned to the intervention group, helping users in their own language. On the day of delivery of the tablet, a training session is held for both the subject and the caregiver, with explanations of how the tablet and the app works.

In addition, a paper-based manual is delivered on the introductory meeting as a complement to the verbal introduction. The manual has been developed with an emphasis on the parts of the tablet and app, where users in the feasibility study experienced difficulties.

Finally, throughout the clinical trial, participants have a contact person in each site with whom they can consult regarding their doubts and technical problems.

The hours and limits of responsibility of this support will be standardized across trial sites. The support will be delivered at clinical visits or via email or phone calls.

### Control Group

The control group will only receive their TAU. There are different conditions and diseases and the treatment can vary widely among the participants. In addition, there are different health care systems among study centers. In any case, the participants in the control group will receive the TAU that they would receive at their care center regardless of the study.

### Outcomes in Persons With Mild Cognitive Impairment

#### Primary Outcome

##### Health Related Quality of Life

Health related quality of life of PwMCI will be measured using the total score of the QoL-AD questionnaire [33,38,39]. This is a 13-item measure, which has been specifically designed to measure QoL in individuals with dementia from the perspective of both the PwMCI and the informal carer. It includes questions related to the interpersonal, environmental, functional, physical, and psychological status of the person with dementia, and thus, it is a global measure for QoL. QoL-AD will be assessed via an interview with PwMCI and via self-completion by informal carers.

#### Secondary Outcomes:

##### Adherence to Prescribed Medication: Doses and Pill Count

Adherence to prescribed medication will be assessed by comparing the PwMCI’s documented prescription for medications with the number of pills taken (or if medication is not in pill form, pill equivalents) in the 30 days before the assessment day.

This assessment will be undertaken by a Principal Investigator PI or delegate at the 6-, 12- and 18-month visits. PwMCI and informal carer dyads will be asked to bring any documentation related to their prescription history for all medications and all the remaining pills (or pill equivalents) and empty medication packaging they have from the previous 30 days so that the researcher can undertake the medication count.

The dose/pill count is the number of pills or doses taken divided by the number of pills or doses prescribed, multiplied by 100 (expressed as a percentage) [40,41]. According to Haynes et al recommendations, a good adherence is considered when the result of counting is between 80% (a 20% of doses/pills missed) and 110% (the patient takes 10% more doses/pills) of doses/pills prescribed. We will select a maximum of 2 drugs for each participant in the pill count.

##### Cognitive Function

It will be measured by the MMSE [42]. It is also used to estimate the severity and progression of cognitive impairment and to follow the course of cognitive changes in an individual over time. To be included in the trial, individuals must score between 20 and 28 points on the scale. The use of an MMSE cutoff value of 28 is not common and has some risks but has been used in other studies [43]. O’Bryant et al [44] showed that an MMSE cutoff score of 28 gave the best sensitivity and specificity for detecting mild dementia in a population with self-reported memory complaints.

##### Functional Decline

The EuroQol-5D (EQ-5D) is a self-completion questionnaire that consists of 5 questions plus a scale where the participant rates their health state on a scale of 0 to 100. EQ-5D has been shown to correlate well with QoL-AD, indicating that the 2 measures are compatible and can be used side by side [38].

##### Service Utilization

Attendance at health care appointments and admissions to health care institutions will be collected from health management systems in the participating regions.

#### Independent Variables-Covariables

##### Sociodemographic Data

Age, gender, education, whether participant lives alone, marital status, and relationship with the informal carer are the sociodemographic variables.

##### Medical History of Persons With Mild Cognitive Impairment

These include family antecedents such as Alzheimer disease, Parkinson disease, other dementing illness; Comorbidity International Classification of Diseases-10; Current Treatment according to The Anatomical Therapeutic Chemical Classification System Information about dementia: diagnosis of dementia, type of dementia, if they have undergone a magnetic resonance imaging scan, and if they are using any pharmacological treatment for their dementia.

##### Depression

The GDS-15 [37] will be used as an exclusion criterion, screening for depression. If participants score above 11, they will be excluded from the trial.

The GDS is commonly used as a routine part of a comprehensive geriatric assessment. The grid sets a range of 0 to 4 as “normal,” 5 to 8 as “mildly depressed,” 9 to 11 as “moderately depressed,” and 12 to 15 as “severely depressed.”

##### Familiarity With Comparable Technological Devices

This will be assessed via a questionnaire of 6 questions tailored to this study, covering both prior and current experience of using internet and mobile technology in general and, specifically, apps with particular relevance to SMART4MD functionality (Multimedia Appendix 2). It will also include an instrument to measure general attitudes and feelings toward technology [45].

##### User Behavior

In the intervention group, the following data will be collected from the platform: date and time of any interaction with the SMART4MD app, length of interaction, and the functions used during the interaction.

### Outcomes in Carers

#### Secondary Outcomes

##### Carer Burden

The Zarit Caregiver Burden Interview (ZBI-12) [46,47] will be used to evaluate informal carers’ burden. The ZBI-12 is a 12-item scale with each answer chosen from a 5-point Likert scale. It is a shortened version of the original scale that was developed specifically for informal carers of PwMCI and covers issues such as carer stress and the degree to which caring is affecting their health and social life. It will be administered via interview with the informal carer.

#### Independent Variables-Covariables

##### Sociodemographic Data

Age, gender, education, whether participant lives alone, marital status, and familiarity with comparable technological devices are the sociodemographic variables.

### Follow-Up Visits

There will be 3 follow-up visits at 6, 12, and 18 months after baseline interview. A summary of visits’ assessments is shown in Table 1. In addition, to measure medication adherence, there will be a preadherence phone call in each follow-up visit (30 days before the visit).

**Table 1:** Schedule of events.

Events, assessments, and data collection	Screening	Baseline	Tablet delivery	Month 6 ±18 days	Month 12 ±18 days	Month 18 ±18 days
Informed consent (PwMCI^a^ and informal carer)	X^b^	—^c^	—	—	—	—
Review of inclusion and exclusion criteria (PwMCI)	X	—	—	—	—	—
Demography (PwMCI and informal carer) and medical history (PwMCI)	—	X	—	—	—	—
Introduction and training for Support Monitoring and Reminder for Mild Dementia (intervention group only, PwMCI and informal carer)	—	—	X	—	—	—
Geriatric Depression Scale (PwMCI)	X	—	—	X	X	X
Mini-Mental State Examination (PwMCI)	X	—	—	X	X	X
Quality of Life in Alzheimer's Disease scale (PwMCI)	—	X	—	X	X	X
Instrumental Activities of Daily Living (PwMCI)	—	X	—	X	X	X
Adherence to prescribed medication (PwMCI)	—	X	—	X	X	X
EuroQol-5D (PwMCI and informal carer)	—	X	—	X	X	X
Attendance at appointments and admissions (PwMCI)	—	X	—	X	X	X
Familiarity with tech survey (PwMCI and informal carer)	—	—	X	—	—	—
QoL-AD on behalf of PwMCI (informal carer)	—	X	—	X	X	X
Zarit Caregiver Burden Interview (informal carer)	—	X	—	X	X	X
User satisfaction survey (intervention group only, PwMCI, and informal carer)	—	—	—	X	X	X
User behavior (intervention group only, informal carer, and PwMCI)	—	—	—	X	X	X

^a^PwMCI: persons with mild cognitive impairment.

^b^The instrument is used at this time point.

^c^The instrument is not used at this time point.

### Statistical Analysis

All analyses will be performed with the SPSS 25+ program (IBM Corp) [48].

#### Summary of Baseline Data and Persons With Mild Cognitive Impairment Progress Through the Trial

For continuous variables we will collect the mean, median, minima and maxima, lower and upper quartiles, and SD. Categorical variables will be summarized using counts and percentages. The progress of the PwMCI will be shown schematically with counts and percentages in a CONSORT diagram. The analysis will adopt the intention-to-treat principle.

#### Primary Outcome Analysis

The primary analysis will be the comparison of total QoL-AD score mean changes (at 18 months) between intervention group and control group using a 2-sample, 2-sided Monte Carlo permutation *t* test. The use of a permutation test will avoid the need for strong assumptions about the distribution of the data. Similarly, a bootstrap approach will be used to obtain the 95% confidence limits for the difference between the group means.

#### Secondary Outcome Analyses

The comparison between intervention group and control group for continuous variables will be analyzed with the same method described above, and categorical variables will be analyzed using Fisher exact test.

Regression analysis will be used to assess the relationship between the primary and secondary outcome measures and the Technology Familiarity Score to analyze whether prior experience of technology has an effect on the outcomes. Furthermore, a regression analysis will be used to assess the relationship between the primary and secondary outcome variables and the usage variables to indicate which aspects of the computer tablet use most affected the outcomes.

A user-behavior analysis will be performed to assess how users interact with the platform and how their behavior affects its efficacy. Specifically, we will assess and analyze the frequency of access to the SMART4MD app, the length of this interaction, and the quality of the inputs provided. These data will be correlated with users’ reminder schedules to explore differences between proactive and reactive use of the platform and will be followed over time to understand how increasing familiarity with the platform affects user behavior. In addition, the data will be used to improve the SMART4MD app, if necessary, to optimize user interaction. The results of the usability tests will be analyzed using statistical methods to quantify the error rate, effectiveness, and learning curve of the SMART4MD app and functions. For this, the intervention group will be asked to perform specific tasks with the app, and different interaction parameters will be measured such as number of taps, reaction time, time to target, time to accomplish, etc.

#### Subgroup Outcome Analyses

Checks for the consistency of the intervention effects across sites will be done using a 2-way analysis of variance with the 2 factors being group (intervention/control) and site. Similarly, a subgroup analysis will be carried out according to the severity of cognitive impairment. These subgroups will be formed based on the MMSE score corrected by educational level and age.

#### Adjusted Analyses

A secondary analysis will be an analysis of covariance to compare the 18-month total QoL-AD score means, where the response variable will be the 18-month total QoL-AD score and the covariate will be the baseline total QoL-AD score. This analysis will provide a comparison of the 18-month total QoL-AD score means adjusted for the baseline total QoL-AD score. Again, the *P* value will be obtained using a Monte Carlo permutation method, and the 95% confidence limits for the adjusted difference between the means will be obtained using a bootstrap approach.

#### Procedures to Account for Missing or Spurious Data

For analyses involving multiple regression analysis, a multiple imputation approach will be considered and used, if statistically sound, depending on the proportion and pattern of missing values.

#### Methods to Ensure Validity and Quality of Data

Accurate and reliable data collection will be assured by verification and cross-check of the electronic case report form (eCRF) against the investigator´s records (source document verification). Source document verification will be conducted for 5% of data in subjects.

A comprehensive validation check program using front-end checks in the eCRF will verify these data. Discrepancies and queries will be generated accordingly in the eCRF for Web-based resolution by the investigator at the site. In addition, the eCRF data will be reviewed on an ongoing basis for medical and scientific plausibility.

### Ethical Considerations

This study will conform to the principles of the declaration of Helsinki. Ethical approval for this trial was granted by the regional ethical review boards at each participating site ensuring full compliance with all research and legislative regulations in the respective countries. The major ethical considerations for this study with respect to the group with PwMCI have been analyzed in depth (Multimedia Appendix 3).

The database used for the unidentified clinical data is located physically at Blekinge Institute of Technology and is used for several other clinical studies including the Swedish National Study of Aging and Care following all relevant protocols for data security and integrity. The code key containing the identifier are kept in a locked cupboard on a computer/Universal Serial Bus memory not connected to the internet.

## Results

Inclusion of participants started in December 2017, and recruitment is expected to end in February 2019. Furthermore, there will be 3 follow-up visits at 6, 12, and 18 months after the baseline visit.

## Discussion

The benefits of this RCT will be at several levels including in-depth knowledge of the possibilities of introducing a holistic multilayered ICT solution for this particular group. Support for the hypothesis that particular SMART4MD functions may improve QoL In PwMCI can be found in other studies. For example, SMART4MD enables users to record a list of their medication, to receive reminders to take their medication, and to record that they have done so. In this way, it encourages treatment adherence. An association between treatment adherence in Alzheimer disease and improved QoL for persons with dementia is claimed in a nonsystematic review of literature on factors affecting adherence to cholinesterase inhibitors, the main class of therapeutic drugs [35]. More specifically, 1 study [49] (the first to address this specific issue) found that people taking acetylcholinesterase inhibitors rated their QoL (using the total QoL-AD score) more highly than those who did not. Similarly, the control of dementia risk factors has consistent evidence for dementia prevention, especially in those at risk of developing dementia because of memory claims and/or MCI. Therefore, the adequate control of chronic diseases, such as hypertension, diabetes, and hypercholesteremia, for which an adequate adherence to pharmacological treatment is necessary, could imply an improvement in the QoL [50].

SMART4MD also enables users to access information about dementia and MCI. A systematic review of literature on information services for persons with dementia and informal carers [51] found that 2 out of the 3 RCTs measuring the QoL of the person with dementia under these circumstances indicated benefit. A systematic review of literature on social support group interventions for person with dementia identified 2 studies, 1 of which showed the QoL benefit of a support group providing educational seminars and supportive discussion on medical causes and treatments and future planning and strategies for enhancing communication and daily living [52,53].

The secondary objectives of the trial comprise establishing possible improvements in medication adherence, functional status, and informal carers’ well-being as a result of using SMART4MD.

The proposition that particular SMART4MD functions may promote these outcomes is also supported in literature. Reviews demonstrating that oral acetylcholinesterase inhibitors improve performance in activities of daily living [54,55] mean that the medication reminder function is well placed to support functional status. Other studies have shown that digital games can support communication and a sense of well-being in persons with dementia [56] and that multimedia memory aids can promote recall [57]. A recent systematic review has also shown some positive results for computerized cognitive training in population with MCI or dementia [58]. Providing information about dementia to informal carers as well as supporting them in mobilizing social support networks have been shown to reduce informal carers´ burden [59]. There is also a significant burden in informal caregivers of individuals with MCI [60]. Assistance with medication regimes not only has the capacity to improve QoL in person with dementia’s or MCI but also that of informal carers [61].

SMART4MD has been developed from an existing general health management app in a process involving the structured participation of PwMCI, their informal carers, and clinicians. The adoption of SMART4MD by older people faces the challenge of this age group’s relative unfamiliarity with digital devices and services. However, this challenge can also be seen as an opportunity. This research entails developing a digital device, which is specifically tailored to a group of people who are particularly at risk of digital exclusion. In this respect, the research responds to the wider call for the development of digital devices that are accessible and affordable to older people, rather than a source of anxiety [62].

SMART4MD’s combination of a number of functions distinguishes it from other comparable products and services, making it the kind of innovative nonpharmacological intervention that experts in the field have called for [56].

The occurrence of dementia and MCI is a common problem across Europe, but its management varies between countries and even regions. Variations in the commitment of member states to national dementia strategies naturally translates into variations in clinical practice through differences in both funding and priority given to dementia in different countries. More generally, health care systems vary widely between member states and regions, with differing emphasis placed on home, community, primary, and secondary care. These differences are further amplified by varying practice in reimbursement of health care costs between private health care insurers and public authorities; and by different levels of funding available for health care. As a result, the support and treatment available for PwMCI across the European Union varies widely. By including PwMCI, informal carers, and health care professionals from various European countries in this study, we can ascertain whether SMART4MD can support PwMCI and informal carers irrespective of these differences in services and treatments.

## Appendix

Multimedia Appendix 1 The intervention strategy for Support Monitoring and Reminder for Mild Dementia.

Multimedia Appendix 2 Support Monitoring and Reminder for Mild Dementia questions on experience of and familiarity with technology.

Multimedia Appendix 3 Additional brief clarification for the potential ethics issues associated with longer-term trials involving older persons with mild cognitive impairment.

## Abbreviations

ARCTU: Anglia Ruskin Clinical Trials Unit
CONSORT: Consolidated Standards of Reporting Trials
eCRF: electronic case report form
EQ-5D: EuroQoL-5D
GDS-15: Geriatric Depression Scale
ICTs: information and communication technologies
MCI: mild cognitive impairment
MMSE: Mini-Mental State Examination
PwMCI: persons with mild cognitive impairment
QoL: quality of life
RCT: randomized controlled trial
SMART4MD: Support Monitoring and Reminder for Mild Dementia
TAU: treatment as usual
ZBI-12: Zarit Caregiver Burden Interview
