# Supplementary material for: The Effects of the Digital Platform Support Monitoring and Reminder Technology for Mild Dementia (SMART4MD) for People With Mild Cognitive Impairment and Their Informal Carers: Protocol for a Pilot Randomized Controlled Trial
Source: JMIR Res Protoc. 2019 Jun 21;8(6):e13711. doi: 10.2196/13711 (PMC6611150; doi:10.2196/13711)
Supplement: Multimedia Appendix 1 [file resprot_v8i6e13711_app1.pdf]

SMART4MD

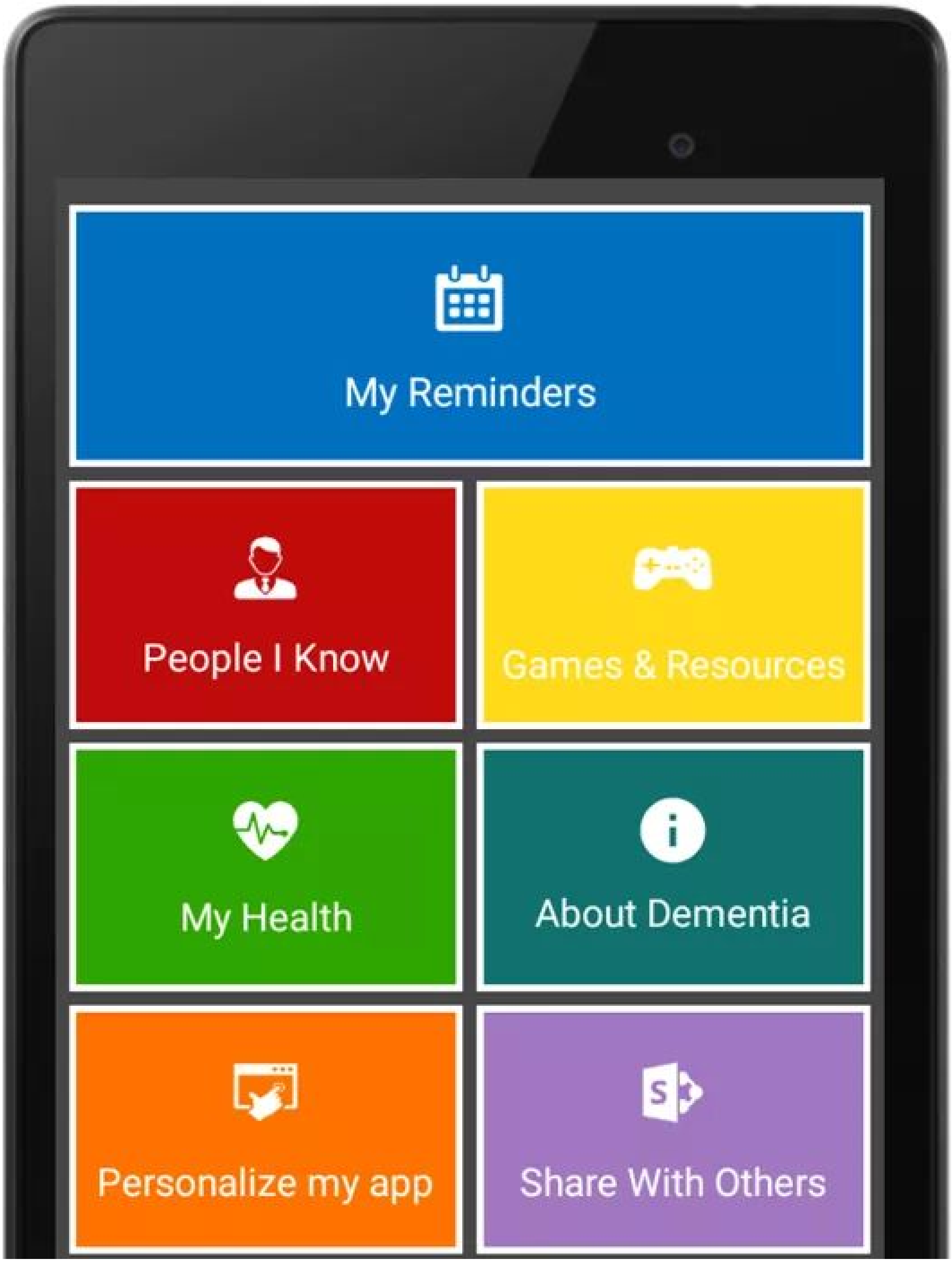
My Reminders
People I Know
Games & Resources
My Health
About Dementia
Personalize my app
Share With Others

The intervention strategy for SMART4MD is explained in detail

The SMART4MD platform (http://www.smart4md.eu/product-tour/) is encompassed within the assistive technologies. Following this line, Khosravi and Ghapanchi (2016) have published a relevant systematic review focused on the use of assistive technologies to improve QoL in older people in general. This covered technology including computer and the internet (i.e., general ICT), robotics, sensors, telemedicine, medication management applications, and video games. Regarding QoL in older people with dementia, specifically, the review identified three studies investigating general ICT and sensors. These suggested that the technologies have a capacity to enhance QoL not only in people with dementia but also in those caring for them (Lauriks et al, 2007; Nugent, 2007).

Recently, results from a randomized controlled pilot study were published using a computerized platform entitled 'A technology platform for the assisted living of Dementia elderly Individuals and their carers' (ALADDIN) (Torkamani et al., 2014). The authors demonstrated that the use of ALADDIN enhanced the carers' ability to care for a person with dementia by improving carer QoL and reducing distress and carer burden.

The scientific literature supports that certain functions included within the SMART4MD application may improve PWDs QoL. These evidences are detailed below:

- Listing and reminders of medication

Many people with dementia often take medication both for dementia itself and for other conditions, thus people with dementia are often prescribed more than one medication (Scrutton et al 2016). One study performed in the UK evidenced, for example, that several residents with dementia were taking an average of eight medicines. On the other hand, prescribing medicine to older people with dementia may be also be challenging "because of the changing needs associated with cognitive decline and related behavioural and psychological symptoms" (Lau et al 2011). Poor medication management may be contributing to people with dementia having worse health outcomes for their comorbidities and may even result in additional comorbidities (Scrutton et al 2016). The SMART4MD application can help to enhance the medication

management of the participants as well as their experience of taking the medication autonomously.

SMART4MD enables users to record a list of their medications, to receive reminders to take their medication and to record that they have done so. In this way it encourages treatment adherence. An association between treatment adherence in Alzheimer's Disease (AD) and improved QoL for PWD is claimed in a non-systematic review of the literature on factors affecting adherence to cholinesterase inhibitors, the main class of therapeutic drugs (Brady and Weinman, 2012). More specifically, one study (Hoe et al., 2007) (the first to address this specific issue) finds that people taking acetylcholinesterase inhibitors rated their QoL (using the total QoL-AD score) more highly than those not, but causality could not be established as this was a cross-sectional study.

In addition, these reminders attempt to improve medication management in subjects with dementia. This instrumental activity of daily life is frequently altered in PWD. It is relevant to carry out interventions that aim to improve the functionality in this population, since a greater independence in activities of daily living is associated with better quality of life in PWD (Chang et al., 2015).

Moreover, in the 2nd round of focus group, conducted in Spain, Sweden, Czech Republic and Belgium in validating the SMART4MD prototype as being usable by people living with mild dementia, showed up the suggestion to add a diary for the person with dementia with notes and images. In this diary PWD could register any side effects or any other difficulties they have experienced whilst taking the medication, so that this could help then to discuss this with doctor and improve medication management.

- Listing and reminders of healthcare appointments and activities

SMART4MD incorporates an agenda where the user can annotate their healthcare appointments and other activities with reminders. These reminders enable people with dementia to maximize their independence through assistive technologies, such as SMART4MD.

On the other hand, the use of reminders in subjects with dementia is a crucial aspect due to the high degree of non-attendance rates. In the study by Dockey et al. (2001), 40% of

the PWD did not attend their healthcare appointment, whereas for subjects without dementia this percentage was 16. In addition, this study showed that the reminders, in this case in the form of calls, were an effective strategy for both the PWD and its caregivers for increasing attendance rates.

Other European projects similar to SMART4MD, such as the European research project COGKNOW (Dröes and Bengtsson, 2009) include this functionality in their assistive technologies. This project was one of the first projects dealing with technology supporting people with mild dementia in Europe. Users and caregivers who participated in COGKNOW project rated the reminder functionality as very useful.

Moreover, an important note is that people with dementia may experience difficulties two remembering such activities. Reminders about activities and hobbies in the SMART4MD app will help participants in this aspect and could then be an important factor in improving their quality of life. Likewise, this could impact on the quality of life and experience of burden of the carer as the technology will assist them in this task.

- Health management

Health management for older people always need to include comorbidities. In a review it was indicated that interventions targeted either at specific combinations of common conditions or at specific problems for patients with multiple conditions, may be more effective (Smith, 2012). The clinical outcomes are improved when chronic disease management programs teaching self-management skills are incorporated rather than information-only patient education and reduce costs (Taylor S, 2014). A systematic review found that self-management interventions are effective in improving both process of care and patient outcomes (Pillay, 2014). The SMART4MD intervention includes the possibility for multiple condition management with the possibility of inclusion of trackers for different chronic conditions.

- Displaying date and time

People with dementia exhibit a wide range of cognitive dysfunctions, among which is the temporal disorientation. For this reason, the date and time will always appear in the SMART4MD application. With this functionality it has the objective of reality orientation. Few systematic reviews are available investigating the effectiveness of reality orientation on the QoL of people with dementia.
The American Psychiatric Association (APA, 2007) has described reality orientation among four different psychotherapeutic approaches that can be useful for treating people suffering from dementia. A recent systematic review (Carrion et al, 2013)

suggests that reality orientation is effective in slightly slowing down functional impairment in people suffering from dementia.

- Cognitive-stimulating activities (games)

Another feature that is included in SMART4MD are cognitive-stimulating activities, like games. A recent systematic review found preliminary evidence that non-pharmacological interventions targeting both patients and their carers (through family carer coping interventions combined with patient activity) improved the QoL of persons with dementia living at home (Cooper et al., 2012). In contrast, a systematic review of pharmacological interventions to improve QoL and wellbeing in persons with dementia found these interventions failed to show positive results in these domains (Cooper et al, 2012b). Within the non-pharmacological interventions, we find the brain games. In recent years, there has been a growth in computer-based cognitive-stimulating activities or challenges. These “games” can be played on a daily basis to “exercise” the brain. Most studies show that mental stimulation was associated with a delay in the presence of cognitive impairment or even cognitive improvement in those subjects who already presented cognitive impairment. More recently, Cheng et al. (2014) show the positive effect of physical and mental activities on delayed cognitive impairment in subjects with dementia. In addition, those subjects who report better quality of life are more likely to benefit from training with games (McDougall and House, 2012).

Participation in cognitive-stimulating activities, especially those involving games such as puzzles and cards, was associated with better performance on cognitive test even with adjustments for education and occupational complexity (Joanitis et al, 2013). Recently, Schultz et al. (2015) found that participation in cognitive activities involving games and puzzles is related to better cognitive abilities and larger volumes in Alzheimer's Disease vulnerable brain structures in a cohort at risk, middle-aged adults. Analysis of data from The Bronx Aging Study cohort assessing the influence of crossword puzzle on persons who developed dementia, showed that this cognitively stimulating leisure activities delayed the onset of accelerated cognitive decline in subjects who developed dementia by 2.54 years compared to non-puzzlers (Pillai et al., 2011).

- Educational content

SMART4MD also enables users to access information about dementia and mild cognitive impairment. A systematic review of literature on information services for PWD and carers (Corbett et al., 2012) found that two out of three randomized control trials measuring PWD’s QoL under these circumstances indicated benefit. A systematic review of literature on social support group interventions for PWD identified two qualifying

studies, one of which showed the QoL benefit of a support group providing educational seminars and supportive discussion on medical causes and treatments, future planning and strategies for enhancing communication and daily living (Lueng et al., 2015).

Another relevant aspect to take into account related to the information on dementia and MCI is that the provision of information can help the person adjust to his/her condition and facilitate access to support and services. A survey carried out in 2006 involving 1,000 carers from five European countries showed that carers often felt that the provision of information on all aspects of Alzheimer's disease was inadequate (Georges et al., 2008). In this study, 19% of the participants received no information at the time of diagnosis and 82% were given no information about available services. Likewise, other small-scale qualitative studies have shown that carers of people with dementia experience difficulties in gaining information about the support and services available to them (Robinson et al., 2009). Research indicates that overall people with dementia and their carers would welcome more information about available support in their local community (Georges et al., 2008; Innes, Szymczynska and Starket 2014; Robinson et al., 2009).

One paper described dementia patient and caregiver outcomes at 12 and 18 months after the start of the intervention that included both education and structured care management (Vickrey et al., 2006). In intervention clinics, they found better health-related quality of life and better overall quality of health care in patients, better informal caregiving quality, better social support and more informal caregivers, who reported receiving all help that they needed.

In this regard, providing caregivers with information concerning their condition and the necessary tools and/or support to cope with stress would bring rewards for both the caregiver and the individual as far as quality of life and health monitoring are concerned (Cheng et al. 2012).

- Social relationships / People

Finally, SMART4MD will also include external aids, like a photo gallery where the PWD may include photos (with name) of family, friends, school friends, etc. In addition, it includes an agenda with the relevant data of the relatives (address, telephone, etc). Previous studies clearly have demonstrated that external aids can be successfully utilized by persons with dementia (Bourgeois et al, 2003).

The WHO (2007) recognizes social participation as a key element for good health and well-being throughout life. Research involving people with dementia has evidenced that maintaining social relationships is relevant to them and that many people with dementia wish to continue doing the activities that they used to do prior to diagnosis (Alzheimer Europe, 2015). However, the onset of cognitive problems may impact the opportunities that people have for social participation and their opportunities to engage with friends and family. Many factors may be relevant to this.

The participants will be provided with a data-enabled computer tablet on which will be installed the SMART4MD application. The tablet will be configured in such a way that it is not possible for participants to download other applications or software onto the tablet.

SMART4MD is a general e-health application which has been adapted specifically for mild dementia and mild cognitive impairment through a structured process involving the participation of users and informal carers. It can run on tablet and mobile phone devices adopting the Android operating system but is specifically optimized for tablet devices as opposed to smaller screen mobile phone devices.

The core functionalities of the application are based on reminders (medication, appointments with healthcare providers), brain supporting activities (clock, calendar, brain games, photos) and optional status and health information sharing with family and informal carers (including daily feeling status, specific health problems, and quality of life). An important feature of SMART4MD is its personalization facility: main users will be able to switch off/on various features and information sharing possibilities.

The application is intended to be used daily at home, mainly by the participants themselves, with the help of their informal carers.

The computer tablets with the SMART4MD application installed must provide only to participants enrolled in the trial in accordance with the protocol.

HealthBit (developer of the application) will provide online support regarding use of the application. In addition, each partner organization will nominate one individual to lead on IT support for participants recruited by that organization and assigned to the intervention group. The hours and limits of responsibility of these local IT support leads

will be defined by the data management steering committee and will thus be standardized across partner organizations.

PWD- informal carer dyads assigned to the intervention group will receive a computer tablet with SMART4MD installed. The tablet will be configured in such a way as to prevent its use for any other purposes. The setting up process and initial personalization of the tablet features will be carried out during the training given to the PWD and informal carer. This process will ensure that the ID number
of the dyad corresponds to the ID number of the tablet. Documentation matching ID numbers to dyads of users will be in the possession of the local investigatory team only, kept in a secure environment, and will not be shared or communicated with the partnership. Following this set-up process, access to the tablet will be via swipe action (so as to avoid having to remember a password) and continued access to the application will be enabled (no need to log-in to the app each time the dyad uses it). During this set up process, an account will be created.

In order to avoid data loss or undesired access from third parties, the computer tablets will be configured so that no other applications can be downloaded, installed or run on the tablets by users.

The Participant Information Sheet will clarify the following:

• The decision of what data to enter onto the computer tablets will rest entirely with the participants
• The decision of whether to intentionally share data entered onto the computer tablets with any parties beyond the research team, for example with other healthcare professionals or family members will also rest entirely with the participants
• The default set up is that informal carers and PWD will be able to access one another's' accounts and see any information entered there.
• Informal carers and the PWD will be discouraged from entering data into each other's accounts on each other's' behalf. The extent to which this is occurring will be monitored.
• Data entered into SMART4MD during the course of the trial will by default be deleted at the end of the trail. Participants will be given the opportunity to opt-in to retain this data.

Data stored and transfer

HealthBit is registered with the Information Commissioner's Office (ICO) under the Data Protection Act. It has a Comprehensive Information Security Policy. Encrypted data is stored within a secure Cable & Wireless data centre in the UK. Access to the Network Operations Centre (NOC) and server cage environment are maintained by a security access system controlled by the data centre ensuring strict access control.

Databases and backups are encrypted using MS and Oracle Transparent Data Encryption (TDE). Source code is scanned using VisualCodeGrepper (VCG) recommended by the Open Web Application Security Project (OWASP).

There will be minimal data saved directly to the tablets in order to protect participants. Data is transmitted and stored on a Pow Health server in a secure data-centre based in the UK. Data is rendered within the application from the server. Some minimal components of the application will be stored in the tablets' cache memory (for example page structure) to facilitate loading of pages. In the
unlikely event that users lose Wi-Fi/phone network connection while entering data, any data entered up to that point will be held in the cache memory until connection is possible, at which point it will be synchronized to the server.

Participants will be instructed at enrolment that if they lose their tablet, they should contact Pow Health immediately and also report the loss to their local research team (they will be provided with contact details). If Pow Health is notified that a tablet has been lost, they will immediately remotely lock access to the application. This will render the initial ID invalid. The research team will then provide participants with a new tablet and new ID. The new ID will be assigned to their historical data on Pow Health servers.

At the end of the study, Pow Health will by default not retain any information participants input on the application in the course of the study. Participants will be told about this in the PIS. However if they would like to be able to access this data in the post study period they will be given a time-limited opportunity to opt in at the end of the study.

References

Alzheimer Europe (2015) Dementia in Europe Yearbook "Is Europe becoming more dementia friendly?" Alzheimer Europe, Luxembourg.

American Psychiatric Association: Practice guideline for the treatment of patients with Alzheimer's disease and other dementias. Arlington, American Psychiatric Association, 2007.

Bourgeois MS, Camp C, Rose M, White B, Malone M, Carr J, Rovine M.A comparison of training strategies to enhance use of external aids by persons with dementia.J Commun Disord. 2003 Sep-Oct;36(5):361-78.

Brady, R., Weinman, J., 2013. Adherence to Cholinesterase Inhibitors in Alzheimer's Disease: A Review. Dementia and Geriatric Cognitive Disorders 35, pp.348–360.

Chan CS, Slaughter SE, Jones CA, Wagg AS..Greater Independence in Activities of Daily Living is Associated with Higher Health-Related Quality of Life Scores in Nursing Home Residents with Dementia.Healthcare (Basel). 2015 Jun 30;3(3):503-18. doi: 10.3390/healthcare3030503.

Cheng, ST, Lau RW, Mak EP, Ng NS, Lam LC, Fung HH, Lai JC, Kwok T, Lee DT. 2012. A benefit-finding intervention for family caregivers of persons with Alzheimer disease: study protocol of a randomized controlled trial. Trials. 2012. Jul 2; 13:98. Doi: 10.1186/1745-6215-13-98.

Cooper, C., Mukadam, N., Katona, C. et al., 2012. Systematic review of the effectiveness of nonpharmacological interventions to improve quality of life of people with dementia. International Psychogeriatrics 24(6), pp.856-870.

Cooper, C., Mukadam, N., Katona, C. et al., 2012b. Systematic review of the effectiveness of pharmacological interventions to improve quality of life and well-being in people with dementia,The American Journal of Geriatric Psychiatry21(2), pp.173-183.

Corbett, A., Stevens, J., Aarsland, D., Day, S., Moniz-Cook, E., Woods, R., Brooker, D., Ballard, C., 2012. Systematic review of services providing information and/or advice to people with dementia and/or their caregivers: Information services for people with dementia and carers. International Journal of Geriatric Psychiatry 27, pp.628–636.

Dockery F, Rajkumar C, Chapman C, Bulpitt C, Nicholl C.The effect of reminder calls in reducing non-attendance rates at care of the elderly clinics.Postgrad Med J. 2001 Jan;77(903):37-9.

Hoe, J., Katona, C., Orrell, M., Livingston, G., 2007. Quality of life in dementia: care recipient and caregiver perceptions of quality of life in dementia: the LASER-AD study. International Journal of Geriatric Psychiatry 22, pp.1031–1036.

Jonaitis E, La Rue A, Mueller KD, Koscik RL, Hermann B, Sager MA. Cognitive activities and cognitive performance in middle-aged adults at risk for Alzheimer's disease. Psychology and Aging. 2013; 28(4):1004–1014

Khosravi, P. and Ghapanchi, A.H., 2016. Investigating the effectiveness of technologies applied to assist seniors: A systematic literature review. International Journal of Medical Informatics, 85(1), pp.17-26.

Lauriks S, Reinersmann A, Van der Roest HG, Meiland FJ, Davies RJ, Moelaert F, Mulvenna MD, Nugent CD, Dröes RM.Review of ICT-based services for identified unmet needs in people with dementia. Ageing Res Rev. 2007 Oct;6(3):223-46. Review.

Leung, P., Orrell, M., Orgeta, V., 2015. Social support group interventions in people with dementia and mild cognitive impairment: a systematic review of the literature: Social support groups in dementia. International Journal of Geriatric Psychiatry 30, pp.1–9.

McDougall S, House B.Brain training in older adults: evidence of transfer to memory span performance and pseudo-Mattheweffects.Neuropsychol Dev Cogn B Aging Neuropsychol Cogn. 2012;19(1-2):195-221. doi: 10.1080/13825585.2011.640656. Epub 2012 Jan 17.

Nugent, CD. ICT in elderly and dementia. Aging Ment Health. 2007 Sep; 11(5): 473-6

Pillai JA, Hall CB, Dickson DW, Buschke H, Lipton RB, Verghese J. Association of Crossword Puzzle Participation with Memory Decline in Persons Who Develop Dementia Journal of the International Neuropsychological Society (2011), 17, 1006–1013.

Schultz SA, Larson J, Oh J, Koscik R, Dowling MN, Gallagher CL, Carlsson CM, Rowley HA, Bendlin BB, Asthana S,, Hermann BP, Johnson SC, Sager M, LaRue A, Okonkwo OC. Participation in cognitively-stimulating activities is associated with brain structure and cognitive function in preclinical Alzheimer’s disease.Brain Imaging Behav. 2015 December ; 9(4): 729–736. doi:10.1007/s11682-014-9329-5.

Torkamani, M., McDonald, L., Aguayo, I.S., Kanios, C., Katsanou, M.N., Madeley, L., Limousin, P.D., Lees, A.J.,Haritou, M. and Jahanshahi, M., 2014. A randomized controlled pilot study to evaluate a technology platform for the assisted living of people with dementia and their carers. Journal of Alzheimer's Disease, 41(2), pp.515- 523.

Vickrey BG, Mittman BS, Connor KI, et al. 2006. The effect of a disease management intervention on quality and outcomes of dementia care: a randomized, controlled trial. Ann Intern Med 145(10): 713–726.
