# Supplementary material for: The Effects of the Digital Platform Support Monitoring and Reminder Technology for Mild Dementia (SMART4MD) for People With Mild Cognitive Impairment and Their Informal Carers: Protocol for a Pilot Randomized Controlled Trial
Source: JMIR Res Protoc. 2019 Jun 21;8(6):e13711. doi: 10.2196/13711 (PMC6611150; doi:10.2196/13711)
Supplement: Multimedia Appendix 2 [file resprot_v8i6e13711_app2.pdf]

Smart4MD questions on experience of and familiarity with technology. Adapted from Eurostat investigation of Internet use by individuals in Europe.

"Do you have access to internet from home?"
Y/N

A1. On average how often would you say you have been using a smartphone or tablet during the last three months?
/Almost every day
/At least once a week, but not every day
/Less than once a week
/Not at all
/I have never used a smartphone or tablet

A2. How often do you use the Internet on your smartphone or tablet?
/Almost every day
/At least once a week, but not every day
/Less than once a week
/I never use the Internet on my smartphone or tablet

A3. How knowledgeable do you consider yourself when it comes to using a smartphone or a tablet?
/Not at all knowledgeable
/Not very knowledgeable
/Quite knowledgeable
/Very knowledgeable

A4. Are you using your mobile phone or tablet as a way to support your memory today?
Y/N

A5. Do you have any special app or software on your mobile phone or tablet that you use to support your memory?
Y/N
If you answered yes to the question above, do you know what the app or program is called?

A6. Do you think that using your mobile phone or tablet to support your memory, helps you to remember things?
Y/N
