# Supplementary material for: The Effects of the Digital Platform Support Monitoring and Reminder Technology for Mild Dementia (SMART4MD) for People With Mild Cognitive Impairment and Their Informal Carers: Protocol for a Pilot Randomized Controlled Trial
Source: JMIR Res Protoc. 2019 Jun 21;8(6):e13711. doi: 10.2196/13711 (PMC6611150; doi:10.2196/13711)
Supplement: Multimedia Appendix 3 [file resprot_v8i6e13711_app3.pdf]

SMART4MD

Additional brief clarification for the potential ethics issues associated with longer-term trials involving older persons with MCI

Johan Sanmartin Berglund - BTH Peter Anderberg - BTH

Table of Contents

Consent...2
Consent process for people with mild dementia...3
Consent process for informal carers...3
Withdrawal and exclusion from the study...3
Strategies to overcome dropouts...4
Description of exit...4
Ethical considerations/potential ethical issues...5
Data protection and participants' confidentiality...5
Carer burden...5
Technical issues and personalisation...6

Consent

A key condition for the conduct of ethical research is that participants have been informed of and understand the purpose of the study and of possible harm which might arise as a result of participation, that they have given informed consent and that appropriate measures have been taken to minimise the likelihood of harm occurring.
Consent must be obtained from PWMCI participating in the trial and the individuals who act as their informal carer.

Consent will only be considered valid if it has been given by a person with the necessary capacity and provided voluntarily (not obtained under duress), based on the provision of relevant information (e.g., full details of what is involved, including possible risks and benefits). To this end, participants will be provided with the information they need to make an informed decision via a Participant Information Sheet. Participants will be given a cooling off period of at least 24 hours between informally agreeing to participate and being invited to formally consent in a meeting with the research team.

A standard Consent Form and Participant Information Sheet will be provided in English for all clinical partners, detailing the list of items to be consented. These and other supporting documents will be prepared by Anglia Ruskin University in consultation with Alzheimer Europe as guidance documents. Each clinical partner will be responsible for ensuring that the consent process they apply locally meets all necessary standards, including translation to local language.

Consent process for people with mild dementia and mild cognitive impairments

Consent is understood in this context as a ‘process’ rather than an ‘event’. Investigators will therefore actively seek reaffirmed consent on all the occasions they seek data from PWMCI. Investigators will use a two-stage test for capacity at recruitment stage and at each subsequent occasion before a participant takes part in a trial procedure or set of trial procedures. Supporting documents supplied to all clinical partners will include a form guiding investigators through this process[1]. The process involves explaining the trial and then carrying out the 2-stage test for capacity. Any investigator gaining consent must demonstrably be qualified to assess the PWMCI’s capacity to consent.

The first stage of the two-stage process is to be sure that the PWMCI is aware of the conditions of participation in the study when giving the initial informed consent. The second stage is to follow up for each visit that the PWMCI is still aware of the conditions of participating taking into consideration the progression of dementia by follow-up questions. This is assessed by the personnel and decided by the PI of the respective study site. If a PWMCI loses capacity to continue participation in the study due to progression of dementia, the legal representative of the PWMCI can decide to withdraw the consent on behalf of the PWMCI. If the legal representative does take over the consent process from the PWMCI, this must be recorded on the trial database by the investigator concerned.

Consent process for informal carers

Investigators must gain consent from informal carers at the start of the trial using the prescribed consent process but do not need to regain consent on each occasion of a trial procedure. However, if a new individual becomes the informal carer of the PWMCI during the trial period, consent must be obtained from that person before they can become involved in the trial.

Withdrawal and exclusion from the study

Participants can withdraw or be excluded from the study for the following reasons:

- Decision to withdraw consent by participant (PWMCI) or by their legal representative (if PWMCI has lost the capacity to consent)[2]
- Death of PWMCI or onset of any physical or mental condition which makes it impossible for them to continue in the opinion of the clinician responsible
- Participants’ tablet is lost or broken and the responsible organisation is unable to replace or repair the tablet
- PWMCI no longer has any informal carer willing to participate in the study. If an informal carer quits, a new informal carer is needed for further participation in the

study. If there is no new informal carer to recruit to the study, the PWMCI can no longer be included and the collection of data ends. Data collected before discontinuation will be used in the analyses.

- Clinical site closes and the PWMCI is unable to transfer to another clinical site.
- Participants excluded due to violation of terms in the tablet contract.

Strategies to overcome dropouts

In order to prevent dropout, a feasibility study will be made to optimize clinical instruments for use in the full pilot, as well as to optimise the usability of the SMART4MD application. To prevent dropouts, encouraging contact with the people in the study will be made throughout the pilot. An example of this could be sending Christmas cards and updating the home page regularly, and other measures necessary to create a commitment to the study. All dropouts will be analysed by the research team and preventive measures will be made to minimise further dropouts.

Description of exit

If a carer quits, a new career is needed for further participation in the study. If there is no new carer to recruit to the study, the PWMCI can no longer be included and the collection of data ends. Data collected before discontinuation will be used in the analyses

If a PWMCI ends his or her participation in the study, the collection of data ends. Data collected before discontinuation will be used in the analyses. If the PWMCI chooses to withdraw from the trial between or during scheduled face-to-face visits, he or she will be encouraged to complete the withdrawal portion of their consent form and send or give it to a member of the research team (they will have been provided with contact details on their consent form). If a dyad is excluded from the trial, the dyad will be contacted by a member of the research team and given an explanation. The date of last contact will be recorded in the informed consent and a copy of the updated document will be given or sent to the former participant.

The data collected until the time of withdrawal or exclusion will be retained in the study (this will have been explained to them on the participant information sheet, during the consent process).

For participants belonging to the intervention group the tablet will be collected by the clinical research team and the return of the tablet will be documented in the Agreement of usage of tablet. A copy of the updated document will be given to the former participants.

Ethical considerations/potential ethical issues

Data protection and participants' confidentiality

The Investigators will ensure that the trial sites comply with the participant confidentiality provisions and privacy laws of each EU member state in which they are domiciled, local regulations, and institutional requirements.

Participants will own and centralise all of their health information within the application. Participants consent to who has access to their information and what information can be viewed. The application creates an audit trail and makes this visible to participants, so they can see who viewed their record, what parts they viewed and from where they viewed it and report any unauthorized access.

The Investigator must ensure that the participant's anonymity is maintained. On the Case Report Forms (CRFs) or other documents submitted to the sponsor or lead clinical site, participants will be identified by a unique identifier. Documents that are not for submission to the sponsor or lead clinical site, (e.g., signed Informed Consent Forms [ICFs]) must be kept in strict confidence by the Investigator.

All data used in the analysis and summary of this study will be anonymous, and without reference to specific study participant names. Access to study participant files will be limited to authorised personnel of the sponsor, the Investigator, and research staff. Authorised regulatory personnel have the right to inspect and copy all records pertinent to this study, but all efforts must be made to remove participants' personal data.

Certain questions in the instruments used in the study might be considered sensitive to reveal, such as QoL-AD (e.g., relationship with family members; financial situation, etc.). Confidentiality is maintained by asking such parts of the interview separately to the PWMCI and informal carer respectively. Further, both PWMCI and informal carers are asked if there is anything in particular they want to share with the clinical test leader or if there is specific information not to be shared with the other.

Carer burden

If the informal carer finds themselves in a caring situation or a level of carer burden which makes further participation in the study difficult or impossible, there are ways of safeguarding the carer's wellbeing. The participants can experience stress and affected mental health due to the use of the tablet and application. An example of this could be increased carer burden for the informal caregiver. In the case this happens, the investigator and their team are responsible for detecting, recording, reporting and taking appropriate actions in according to the requirements of the local institutional review board (IRB) and the appropriate regulatory body (IES) with health- and social care in each country of the study.

Technical issues and personalisation

In order to prevent technical issues with the tablet and application, a feasibility study will be conducted before the full pilot to solve potential technical issues. Additionally, local technical support will be given by appointed persons at each site throughout the pilot to give guidance on the usage of the application and to solve technical issues. If technical issues cannot be solved by the local technical support, POW Health will be contacted for further support. All technical issues and suggestions of improvements will be reported continuously to POW Health in order to improve the application. In the M6 and M12 visits, help with personalisation of application is offered by the clinical test leaders at each site.
